# Dietl's Crises

**Published:** 1908-06-20

**Authors:** 


### Medicine.

### DIETL'S CRISES.

It is probably agreed by most clinicians, that in many cases in which the symptoms consist of indefinite pains and aches, and at the same time the only objective abnormality is undue mobility of the right kidney, the trouble is not really renal. Many of the pains have their origin at the cerebral or spinal rather than at the renal ends of the patient's nerves. Nevertheless, there are a few cases in which mobility of the kidney is directly responsible for acute attacks of pain, as severe as that of renal colic, and often accompanied by hæmaturia. Such paroxysmal attacks of renal colic, due to a movable kidney, are known as Dietl's Crisis, and the following are two examples of the condition:—

The first was the case of a 'bus conductor aged forty-two. Upon three separate occasions during the last year, and each time when he was upon the top of his omnibus, upon a road where the vehicle was constantly lurching, he had been suddenly attacked by terrific pain in his right flank, radiating forwards and downwards towards his right thigh and testicle. The pain was so severe upon each occasion, that he had to clutch the rail to prevent himself from falling, and he had to be helped down and conveyed home to bed in a cab. The pain was usually much less by next day, though once he was incapacitated for a week.

Upon examination in hospital, he seemed to be a very healthy and robust man. His only abnormalities were a freely movable right kidney, and a fulness or resistance over the region of his cæcum. The urine was perfectly normal, and there had been no hæmaturia; the *x*-rays indicated no calculus. From his age, and the recent onset of symptoms, it was thought that the movable kidney could have nothing to do with the pains, and that appendicular adhesions were more probably their cause. So bad had they been, that the patient desired an operation, and in the first instance the appendicular incision was made. The vermiform appendix was, however, absolutely healthy and free from adhesions. The cæcum was not diseased, and no cause for the apparent fulness in this region was found. The right kidney, on the other hand, was so freely movable that it could be drawn down as far as the pelvic brim, and pushed right up under the ribs. A second incision was therefore made, this time in the loin, and the kidney was stitched into place. The organ itself was perfectly sound, but there was an aberrant renal vein entering the lower pole of the kidney, and causing a kinking of the ureter across it whenever a sudden muscular effort of the diaphragm drove the upper pole of the kidney forwards and downwards. The sudden effort made by the patient to retain his balance, when the omnibus lurched severely, was obviously the primary cause of the kinking of the ureter across the aberrant vessel in this way. The patient made an excellent recovery, and continued his omnibus work without any recurrence of his colic.

In the second case there was no operation at which to verify the diagnosis, but, clinically, it was a clear instance of Dietl's crisis, accompanied by hæmaturia, and leading to hydronephrosis. The patient was a woman, aged 28, who had two children. She was conveyed to hospital complaining that suddenly the same morning she had been seized with acute pain in the right flank, shooting down into the groin, and accompanied by vomiting. On examining the right side, nothing definite could be made out owing to contraction of the abdominal muscles locally, but there was great tenderness of the right hypochondriac and lumbar regions. The urine contained blood in fair amount, and a corresponding trace of albumen. No renal tube casts, no pus, and no crystals could be found. All the other systems seemed natural, and a diagnosis of renal calculus seemed probable.

Inquiry showed that she had had two similar attacks previously: one eight years before; the other three years ago. Apart from these three acute attacks of renal colic, she had often been troubled by slighter pain in the right side, and she had noticed a swelling there, which came and went, and was always biggest at the times when the pains were worst. She had also noticed that when the swelling disappeared, she invariably passed an increased quantity of urine. The history was typical of intermittent obstruction to the ureter and hydronephrosis. The cause of the acute attack upon the first two ocasions is not known; the third came on when she was scrubbing a floor, and had just lifted a heavy pail full of water.

Blood was still present in the urine, but in rapidly lessening amount, for two days after the attack; and thereafter the urine was perfectly natural. The muscular resistance disappeared, and an enlarged and freely movable kidney could readily be felt in the right hypochondriac and lumbar regions. It could be held between the two hands, and manipulated not only upwards and downwards, but also from side to side, though it was not possible to push it across the middle line. When the tumour was firmly grasped, the patient experienced the typical feeling of sickness.

The diagnosis of hydronephrosis and freely movable kidney was now obvious, but the possibility of renal calculus as well was not absolutely excluded. The patient was carefully skiagraphed, but no sign of stone could be found. The urine was examined for crystals on many occasions, and none of noteworthy size were found. Blood remained absent from the urine.

It is probable that operative fixation would have been the wisest course, but the patient preferred to postpone surgical measures, and returned to her work wearing a renal belt and pad. It is likely that some day she will have a return of her acute renal colic and hæmaturia—in other words she is liable at almost any time, particularly after any sudden or strenuous effort, to another Dietl's crisis.

